# Optimization of Culture Conditions for Some Identified Fungal Species and Stability Profile of **α**-Galactosidase Produced

**DOI:** 10.1155/2013/920759

**Published:** 2013-01-28

**Authors:** A. S. Chauhan, N. Srivastava, H. K. Kehri, B. Sharma

**Affiliations:** ^1^Department of Biochemistry, Faculty of Science, University of Allahabad, Allahabad-211002, Uttar Pradesh, India; ^2^Department of Botany, Faculty of Science, University of Allahabad, Allahabad-211002, Uttar Pradesh, India

## Abstract

Microbial **α**-galactosidase preparations have implications in medicine and in the modification of various agricultural products as well. In this paper, four isolated fungal strains such as AL-3, WF-3, WP-4 and CL-4 from rhizospheric soil identified as *Penicillium glabrum* (AL-3), *Trichoderma evansii* (WF-3), *Lasiodiplodia theobromae* (WP-4) and *Penicillium flavus *(CL-4) based on their morphology and microscopic examinations, are screened for their potential towards **α**-galactosidases production. The culture conditions have been optimized and supplemented with specific carbon substrates (1%, w/v) by using galactose-containing polysaccharides like guar gum (GG), soya casein (SC) and wheat straw (WS). All strains significantly released galactose from GG, showing maximum production of enzyme at 7th day of incubation in rotary shaker (120 rpm) that is 190.3, 174.5, 93.9 and 28.8 U/mL, respectively, followed by SC and WS. The enzyme activity was stable up to 7days at −20°C, then after it declines. This investigation reveals that AL-3 show optimum enzyme activity in guar gum media, whereas WF-3 exhibited greater enzyme stability. Results indicated that the secretion of proteins, enzyme and the stability of enzyme activity varied not only from one strain to another but also differed in their preferences of utilization of different substrates.

## 1. Introduction

The *α*-galactosidases are the group of glycoside hydrolases (glycosidases or carbohydrases) (EC 3.2.1); the enzymes that catalyze hydrolytic cleavage of O-glycoside bond and belong to the enzymes of carbohydrate catabolism. The *α*-galactosidases (EC 3.2.1.22, *α*-D-galactoside galactohydrolase) hydrolyze the terminal *α*-1,6-linked nonreducing *α*-D-galactose residues from linear and branched oligosaccharides and polysaccharides like melibiose, raffinose, stachyose, short fragments of galacto(gluco)mannans, and galactolipid. According to their substrate specificities, *α*-galactosidases can be divided into two groups [1]. The first group contains *α*-galactosidases active only on oligosaccharides with low degree of polymerization, for example melibiose, raffinose, stachyose, and short fragments of galacto(gluco)mannans. These enzymes are usually very active on artificial substrates like p-nitrophenyl-*α*-D-galactopyranosides [2]. The second group of *α*-galactosidases is active on polymeric substrates. However, similar to the enzymes of the first group, they attack short oligosaccharides, mainly fragments of degraded polymers, as well as artificial *α*-galactosides. 

Galactose is found in many different oligo- and polysaccharides which are present in plants and serve as matrix and storage components. The most abundant polymers containing galactose are galactomannans. The amount and distribution of *α*-1,6-D-galactosyl side groups along the *β*-1,4-D-mannopyranose backbone in galactomannans depends on the species of different organisms. For example, guar galactomannan (guar gum, GG) contains 38–40% galactose [3]. Galactoglucomannans are the main group of hemicelluloses in softwoods. In addition, galactose is known to be a component in complex slime and gum substances [4].

Microorganisms are the most promising sources of large-scale enzyme production. They can be easily grown, and it is usually not difficult to scale up the production process. With microbes, it is possible to increase the production by modifying the growth conditions. *α*-galactosidases of microbial origin (bacterial, fungal) have a number of biotechnological applications, like in beet sugar industry; these enzymes are used to remove raffinose from beet molasses and to increase the yield of sucrose [5]. They are also used to improve the gelling properties of galactomannans to be employed as food thickeners [6] and to degrade the raffinose family sugars (raffinose, stachyose, and verbascose) in food and feed materials such as soya milk. Currently, the research interests in *α*-galactosidases have increased because of its varied applications in human medicine as several *α*-galactosidases are able to cleave off the terminal *α*-1,3-linked-D-galactosyl residue from the type-B blood group converting it into blood group-O [7]. Moreover, those *α*-galactosidases, that are able to hydrolyze terminal glyco(sphingo)lipid *α*-galactosyl residue, might be used for treatment of Fabry disease [8, 9] and X-chromosome-linked recessive lysosomal storage disorder. The disease is caused by a deficiency of the lysosomal *α*-galactosidase A, resulting in a progressive accumulation of glycosphingolipids, predominantly globotriaosylceramide, throughout the human body [10]. 

The *α*-galactosidase is widely distributed in microorganisms, plants, and animals [11]. Microorganisms have advantage of being highly active producers of certain industrially important enzymes. Among them, the *α*-galactosidases from filamentous fungi are most suitable for technological applications because of their extracellular localization, acidic pH optimum, and broad stability profiles [12]. Various microorganisms such as fungus [11, 13], yeasts [14], and bacteria [15] produce *α*-galactosidase. It has been documented that several *α*-galactosidases could be translated by *Penicillium ochrochloron, P. purpurogenum *[16], *P. simplicissimum* [17], and *P. brevicompactum* [18]. In addition, abundant information is available on the biosynthesis of *α*-galactosidase from filamentous fungi belonging to the different genera of *Aspergillus* [19–21]. To explore efficient fungal strains from the same genera is still a promising area of investigation. 

 The aim of the present research paper is to isolate potential fungal strains for *α*-galactosidase production from rhizospheric soil of different habitats, identify their specific strains, and to optimize basal liquid culture media conditions in order to induce maximum growth and enzyme production by selecting different carbon sources in standardized media. The total protein secreted by different fungal strains in the culture broth was also monitored along with evaluation of the stability of enzyme activity in culture filtrate initially at the seventh day and then after every 30 days intervals up to three months duration. 

## 2. Materials and Methods 

### 2.1. Chemicals

p-nitrophenyl-*α*-D-galactopyranosides (pNPGal), synthetic substrate for screening of *α*-galactosidase activity, para-nitrophenol (pNP), chromogenic substrate for standard preparation were purchased from Sigma Chemical Co. (St. Louis, MO, USA); Folin-Ciocalteu's phenol reagent and sodium carbonate were from Merck Chemical Supplies (Darmstadt, Germany). All other chemicals used were of analytical grade. 

### 2.2. Isolation of Fungal Strain

Selected fungal strains of* Penicillium glabrum *(AL-3), *Trichoderma evansii* (WF-3),* Lasiodiplodia theobromae* (WP-4), and *Penicillium flavus* (CL-4) were isolated from rhizospheric soil of *Phyllanthus emlica* (Aanwla plant), *Clitoria ternatea* (butterfly pea/aparajita plant), and soil of local garden of Sagar, India. Fungal colony was selected by performing direct plating [22] method on potato dextrose agar (PDA) plates. A pure culture of all four fungal strains was isolated and maintained on separate PDA slants and stored at 4°C. 

### 2.3. Identification of Fungi in Culture

The cultural characteristics of the colonies were observed on potato-dextrose agar (PDA) plates at 28°C for 7 days. Morphological characteristics of conidiogenous cells and conidia were observed by slide culture on the previously mentioned agar media at 28°C for 3 to 7 days. The isolates were identified based on their morphological and cultural characteristics according to the criterions laid down [23–25].

### 2.4. Identification of Fungal Strain Morphologically by Staining

The isolated strains of *Penicillium glabrum* (AL-3), *Trichoderma evansii *(WF-3),* Lasiodiplodia theobromae* (WP-4), and *Penicillium flavus* (CL-4) inoculums were picked up at the fourth day of culture incubation, placed onto a glass slide, stained with a few drops of cotton blue dye (6 *μ*g/mL) by spreading the sporulated fungi with the help of a sterile needle. The stained material was covered with glass cover slip and visualized under the compound microscope to study the morphological characteristics of the organisms.

### 2.5. Optimization of Culture Medium for Optimal Growth of Microorganisms

Three pellets of heavily sporulated fungi from 4 to 5 days old cultures were picked up through cork borer (1 × 1 cm in diameter) and were added to Erlenmeyer flasks (100 mL capacity) containing 50 mL of liquid medium consisting of KH_2_PO_4_ (7.0 g L^−1^), K_2_HPO_4_ (2.0 g L^−1^), MgSO_4_·7H_2_O (0.1 g L^−1^), (NH_4_)_2_SO_4_ (1.0 g L^−1^), yeast extract (0.6 g L^−1^), and 1% (w/v) dry contents of each of the selected substrates such as guar gum (GG), soyabean casein digest (SC), and wheat straw (WS) extract. The cultivation was carried out on rotary shaker (120 rpm) at 28°C. After 7 days of incubation, the mycelium was separated from culture broth by filtration through Whatman filter paper-1, and the supernatant containing partially purified enzyme filtrate was further used for assaying *α*-galactosidase activity.

### 2.6. Enzyme Activity Assay

*α*-galactosidase assay was carried out in test tubes by the modified version of the method by using p-nitrophenyl-*α*-D-galactopyranoside (pNPGal) as substrate. The assay system contained 0.5 mL of 0.05 M sodium acetate buffer (pH 5.0), 0.9 mL of 1.0 mM pNPGal solution, and 100 *μ*L of enzyme preparation. The reaction was started by addition of pNPGal. The reaction mixture was incubated for 10 min at 50°C and was stopped by the addition of 0.5 mL of 1.0 M sodium carbonate solution. The amount of p-nitrophenol (pNP) released was determined spectrophotometrically using UV-Visible double beam spectrophotometer (Spectrascan UV 2700) at 405 nm. One unit (U) of enzyme was defined as the amount of *α*-galactosidase enzyme which liberates 1 *μ*mol of pNP per min under the given assay conditions.

### 2.7. Measurement of Enzyme Activity Secreted into Culture Medium

The activity of enzyme excreted by each of the fungal strains into the culture filtrate was monitored first at the seventh day and then after at the intervals of 30 days up to three months using the assay procedure as described previously.

### 2.8. Measurement of Stability of Enzyme after Storage at −20°C

The aliquots of enzyme excreted by each of the fungal strains utilizing different carbon sources as substrates present into the culture filtrate were collected first at seventh day, assayed for its activity, and stored at −20°C. Then after at every interval of 30 days for three months, the enzyme was assayed for evaluation of the stability of the enzyme activity. 

### 2.9. Protein Estimation

The extracellular protein content excreted in the culture filtrate by each of the fungal strain was determined by the method described by [26]. Using bovine serum albumin (BSA) as a standard, the culture filtrate without any fungal inoculums was used as a control.

## 3. Results and Discussion

The isolated fungal strains AL-3, WF-3, WP-4, and CL-4 were revived from rhizospheric soil of *Phyllanthus emlica* (aanwla) and *Clitoria ternatea* (butterfly pea/aparajita plant) grown in local garden soil of Sagar, India. These strains were further characterized as *Penicillium glabrum, Trichoderma evansii, Lasiodiplodia theobromae,* and *Penicillium, *respectively, on the basis of their morphological examinations as observed under the microscope and culture characteristics.

### 3.1. Identification of Fungi

**Figure 1:**
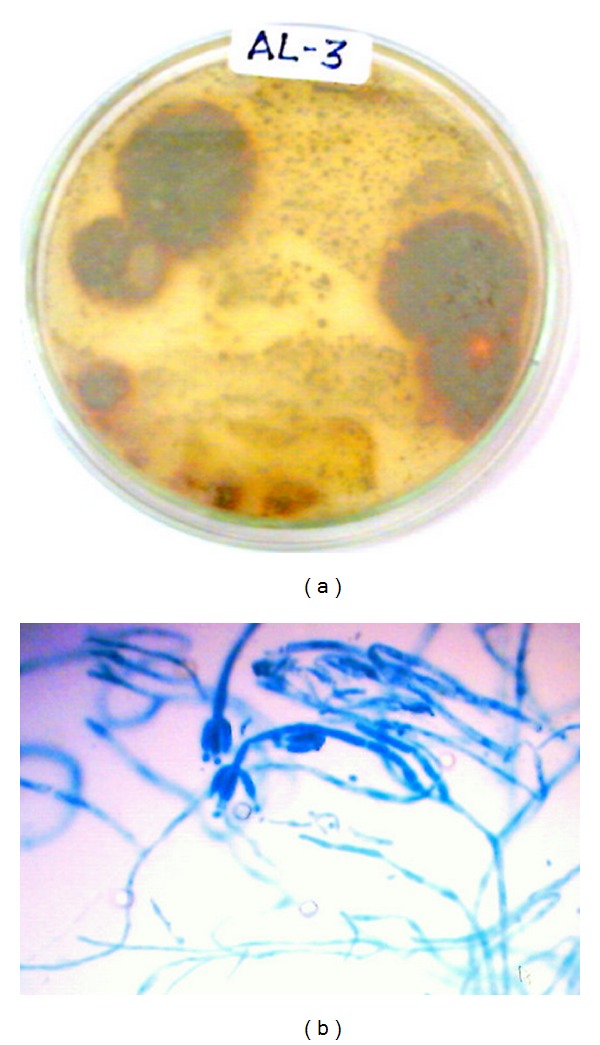
(a) Growth of AL-3 strain after 6 days of growth on culture plate (PDA). (b) Staining photograph of AL-3 strain after fourth day of culture growth on PDA plates.

**Figure 2:**
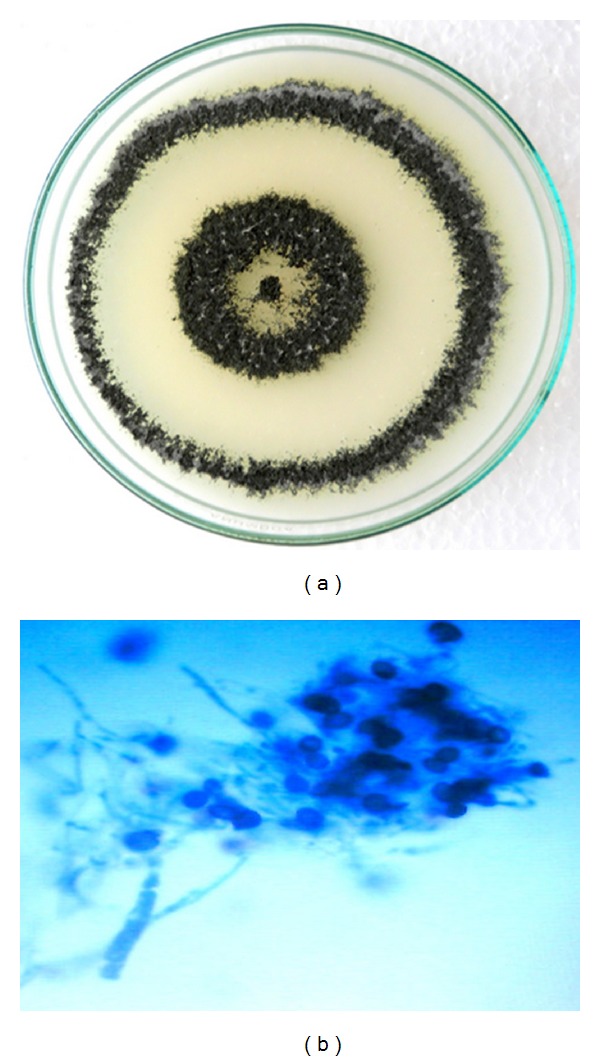
(a) Growth of WF-3 strain after 6 days of growth on culture plate (PDA). (b) Staining photograph of WF-3 strain after fourth day of culture growth on PDA plates.

**Figure 3:**
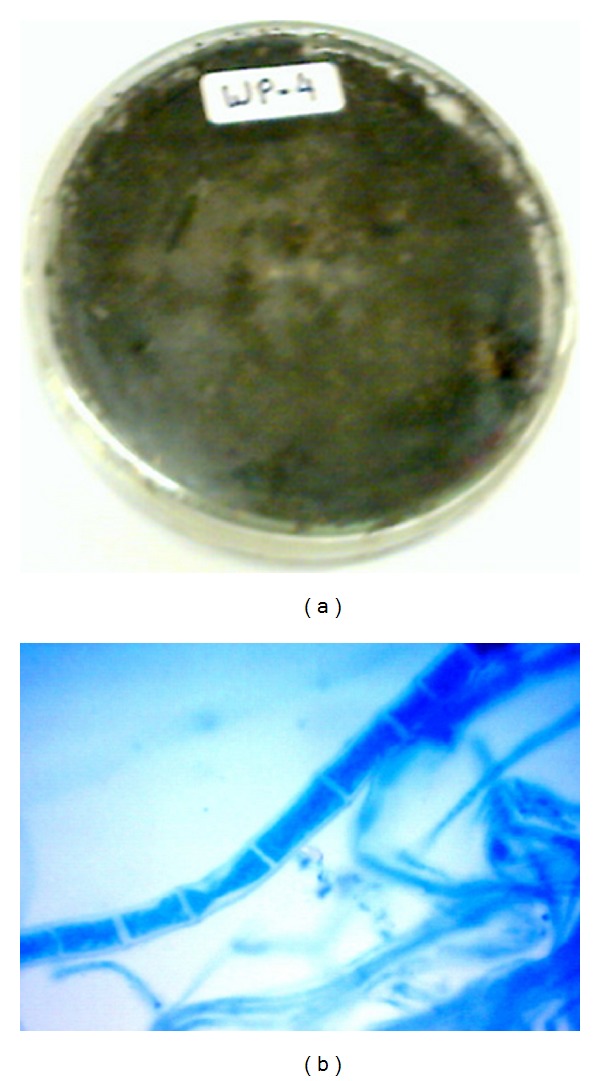
(a) Growth of WP-4 strain after 6 days of growth on culture plate (PDA). (b) Staining photograph of WP-4 strain after fourth day of culture growth on PDA plates.

**Figure 4:**
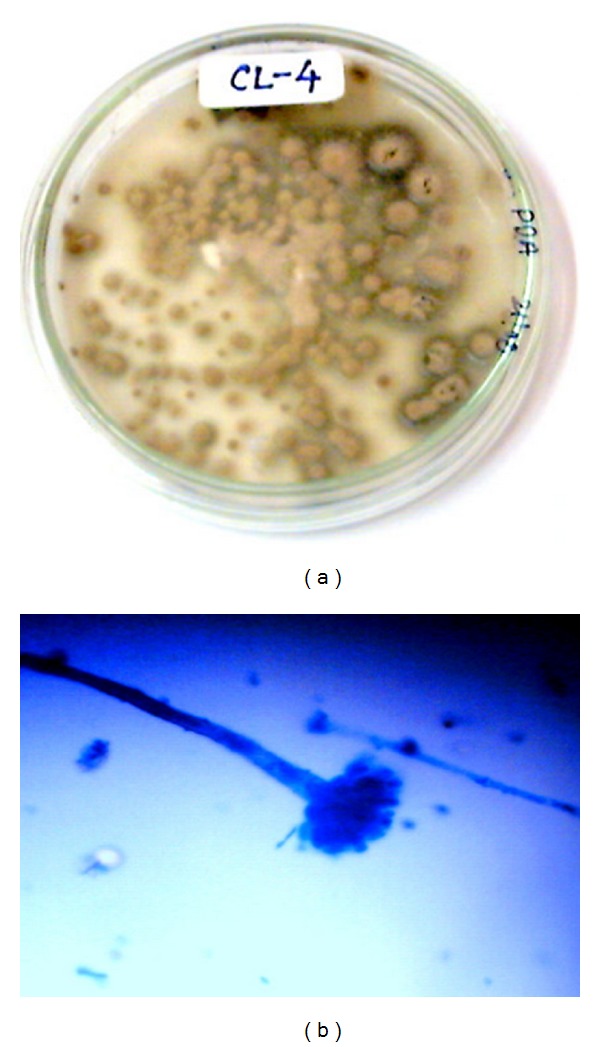
(a) Growth of CL-4 strain after 6 days of growth on culture plate (PDA). (b) Staining photograph of CL-4 strain after fourth day of culture growth on PDA plates.

The isolated fungal strains AL-3, WF-3, WP-4, and CL-4 and their growth on PDA culture plates with mycelial mass after 7 days of incubation at 28°C are shown in Figures 1(a), 2(a), 3(a), and 4(a), respectively. The morphological characteristics as viewed under the microscope are shown in Figures 1(b), 2(b), 3(b), and 4(b), respectively. 

### 3.2. Identification of AL-3 Strain of *Penicillium glabrum*

The mycelial mass of AL-3 after 2 days of incubation showed variation in colour. It initially appeared white and then gradually turns to be green in colour with yellowish pigments at the fifth day when observed from front side, which on maturity becomes orange to dark brown from reverse side (Figure 1(a)) on the seventh day. From the front side, the AL-3 on seventh day showed the colour of mycelia to be greenish brown. The exudates were absent. The mycelia mass of AL-3 on PDA at 28°C appeared slowly reaching to 24–26 mm in diameter by the seventh day. After staining of the mycelia with cotton blue, the identified fungus represented the properties of *Penicillium* genus as it displayed monovertecillate (penicillin typically in single verticils of phialides born on branches which maintained the identity of each vertical). Conidiophores were erect, septate, and branched. Conidia appeared in globuse to ovate in shape and born as 2-3 in chains, which typically forms brush like head (Figure 1(b)). Similar characteristics of Penicillium species have already been reported [27]. Out of three culture media such as guar gum (GG), soybean casein digest (SC) and wheat straw (WS) tested, guar gum (GG) registered optimum fungal growth, microscopically similar to that already reported [27].

### 3.3. Identification of WF-3 Strain of *Trichoderma evansii*

Mycelial mass of WF-3 grown on PDA at 28°C is shown in Figure 2(a). It initially appears with white mycelium on fourth day, forming condia in a central disk of 2 cm diameter and in two pronounced, continuous concentric rings of green conidiophores alternating with rings of sterile, felty, and white mycelium at the end of seventh day and reverse side of plate was observed colorless. No pigmentation or distinctive odor was noted on PDA medium. Colony radius on PDA after 96 h in intermittent light at 28°C was 70 mm with the fungus colony completely filling the petri plate. Similar observations were recorded for the similar strain isolated from elsewhere [28]. After staining of the mycelia with cotton blue, the identified fungus represented the properties of genus *Trichoderma* (Figure 2(b)). Setae arising from the entire pustule were abundant, conspicuous, white, acute at the tip, undulating, septate, infrequently branched, thin-walled, smooth, primarily sterile, and occasionally producing a single, terminal phialide. Fertile branches arising at right angles from the base of setae displayed branches proximal to the tip of the setae typically comprising one or a few cells, terminating in a single phialide or a terminal whorl of 3–5 phialides. Conidiophores were also found to be arising independently of setae (Figure 2(b)). However, the presence of subglobose to globose conidia is unusual in Trichoderma. Conidia of *T. evansii* distinguish it from *T. hamatum* or *T. pubescens*, being found in unrelated species *T. viride*, *T. viridescens,* and *T. atroviride* [29], *T. harzianum*, and *T. aggressivum *[30, 31] and now identified as a new species of *Trichoderma* as *T. evansii* [28].

### 3.4. Identification of WP-4 Strain of *Lasiodiplodia theobromae*

These isolates were obtained from rhizospheric soil of *Clitoria ternatea* (Butterfly pea/Aparajita plant). Its mycelia mass grew fast on PDA at 28°C, took 3-4 days invariably to cover the 90 mm petri plates, and covered the surface of lid in petri plate within 7 days (Figure 3(a)). Mycelial growth pattern is aggregated with fluffy appearance. The color of mycelia colonies was initially light grey which turned into greyish black at later growth stages. All the isolates turned black due to enormous spores production. The reverse side of the colony appeared dark black in color. Sometimes exudation in the form of hyaline drops condensing on the lid of the Petri plates was also observed (Figure 3(a)). After staining of the mycelia with cotton blue, the identified fungus represented the properties of genus *Lasiodiplodia*. Under microscopic examination, matted hyphae was observed to be forming stroma, which contained several pycnidia. The presence of pycnidia was regular, round, flask shaped, situated superficially, or partially immersed in the substrate. The mycelia were septate with big and numerous stroma. Cultural characteristics of WP-4 were similar to LT3a isolate of *Lasiodiplodia theobromae *as reported by other workers [32].

### 3.5. Identification of CL-4 Strain of *Penicillium flavus/Talaromyces flavus*

Mycelial mass of CL-4 grown on PDA at 28°C, initially appeared white on fourth day, later on turns light pink in color without any pigmentation, and exudates appeared on the petri plate. Talaromyces (until now classified in *Penicillium* subgenus *Biverticillium*) have narrower phialides that are aculeate or lanceolate, and anamorphs in *Penicillium* sensu stricto have broader ampulliform or flask-shaped phialides. Vegetative hyphae hyaline to yellow often are encrusted, 1–4 *μ*m in diameter. Ascomata usually yellow, in some strains buff or pinkish to purple-red, globose, 200–700 *μ*m in diameter, commonly confluent but at the margin occasionally discrete, ripening within 2 weeks. Conidiophores arising primarily from the substratum, especially in marginal areas, occasionally are borne also as short branches from aerial hyphae overgrowing the ascomata, usually erect, 24–250 × 1.5–2.5 *μ*m. Metulae 2 to 3 (−4) in the verticil, 10–15 × 1.7–2 *μ*m, occasionally are lacking [33, 34]. 

### 3.6. Effect of Different Carbon Sources on *α*-Galactosidase Production

The effect of different carbon sources on *α*-galactosidase production by different fungal strains tested is depicted in Table 1. When carbon sources were used individually, the maximum enzyme production (190.3 U/mL) was obtained in the presence of 1% w/v guar gum (GG) by isolated fungal strain AL-3 of *Penicillium glabrum*, followed by WF-3 of *Trichoderma evansii *(173.4 U/mL), WP-4 of* Lasiodiplodia theobromae* (93.9 U/mL), and CL-4 of *Penicillium flavus *(63.49 U/mL). Other carbon sources used as substrates were wheat straw (WS) and soya casein (SC) which showed relatively lower enzyme activities at the seventh day of incubation of respective fungal strains culture filtrates. Similar results were obtained by other workers [34] when culture filtrate of *A. fumigatus* was supplemented with 1% (w/v) of galactose, lactose, melibiose, and raffinose. Out of these four carbon sources, galactose proved to be a good inducer for the highest enzyme production (96.70 U/mL) after 2 days of incubation period, followed by melibiose and raffinose. This was in agreement with the results previously reported for the production of *α*-galactosidase by *A. fumigatus* [35, 36], *Trichoderma reesei* [37], and *Penicillium simplicissimum* [38]. Surprisingly, in this investigation, soya casein (SC) sustained substantial growth, but this substrate was almost as poor inducer as lactose [34]. This could be due to presence of invertases, which hydrolyses the soya casein (SC) producing simple sugars in combination with background *α*-galactosidase. These sugars could then be used for the production of mycelia mass but were unable for any further inducing *α*-galactosidase production. 

### 3.7. Effect of Different Carbon Sources on the Level of Total Protein Secreted by Different Fungal Strains in Culture Filtrate

The varying levels of enzyme secreted by different strains of the fungal species tested prompted us to monitor the level of total proteins secreted by these fungal species in the present investigation. The results shown in Table 2. demonstrated that in the presence of GG as substrate, AL-3 could secrete maximum protein (464.5 *μ*g/mL) into the culture medium, whereas the strains WF-3 and WP-4 preferred the presence of WS as substrate to utilize efficiently for the production of maximum excretory protein; the values are 283.5 ± 6.39 and 265.7 ± 5.98, respectively. However, the strain CL-4 preferentially utilized SC as substrate for excretion of protein into the culture filtrate; the value is 277.5 ± 6.08 *μ*g/mL. It is interesting to note that all these fungal species exhibited maximum activities of the secretary *α*-galactosidase in the culture filtrate containing GG as substrate but, they differed in their total protein secretion potential in the medium (Table 2). Similar results have been reported by other labs [34].

### 3.8. Effect of Incubation Time on Stability of Enzyme Present in Culture Filtrate

The effect of different incubation periods on *α*-galactosidase production using basal fermentation medium is shown in Table 3. The optimum production was obtained at the seventh day of incubation period; maximum activity was shown by AL-3 fungal strain, that is, 190.3 U/mL of culture filtrate in guar gum media (GG), followed by WF-3 (173.4 U/mL), WP-4 (93.9 U/mL), and CL-4 (63.49 U/mL), while longer incubation of 30 days showed decreasing trend in enzyme activity; the values are 13.62 U/mL, 76.2 U/mL, 62.9 U/mL and, 25.3 U/mL with AL-3, WF-3, WP-4, and CL-4, respectively. It has already been established that the microbial production of *α*-galactosidase varies with the growth rate [39] and the activity increases with increase in biomass concentration [40]. The growth of the culture increased with the period of incubation; enzyme production also increased simultaneously as shown in preliminary experiments of Anisha and Prema [39], where maximum concentration of enzyme in culture media coincided with the growth of the culture. They have shown that the growth of the culture increased with the period of incubation; the enzyme production also increased accordingly. The highest enzyme production for AGP47 and AGP42 was reported after fourth and sixth day of incubation, respectively; after which cell mass declined and also the enzyme production [39]. Similar to our results, El-Gindy et al. [40] reported that sixth day of incubation was the best for the experimental fungi where *A. awamori *produced maximum *α*-galactosidase activity (2.172 U/g), while *A. carbonarius *reached to maximum *α*-galactosidase production at incubation period of 6 days (2.280 U/g). The activity of *α*-galactosidase secreted by *A. awamori *and* A. carbonarius* showed reducing trend, the values are 1.6 U/mL and 1.8 U/mL, respectively, upon increasing duration of incubation after 7 days. The decline of total enzyme activity could be considered to be the result of inhibition of cellular functions and due to depletion of nutritional factors from the growth medium or deactivation of enzyme due to pH change or due to inducer exclusion. In present investigation, we have found that upon storage of isolated *α*-galactosidase at −20°C, the enzyme activity was stable up to 7 days and then after it declines. The activity remains about 6-7% at the end of the thirtieth day of storage (data not shown).

## 4. Conclusion

The results of the present investigation demonstrate the identification and characterization of four different fungal strains exhibiting potential to secrete *α*-galactosidase maximally when GG was used as carbon source in the culture medium. With other carbon sources (WS and SC), these strains showed relatively lower enzyme secretion potential. The secretion of the enzyme by these fungal strains was maximum at seventh day of the culture with GG as a carbon source. The enzyme activity declines then after, under similar culture conditions. Also, these fungal strains secreted maximum total protein on the seventh day in culture filtrate, but then after there is no change in the total protein secreted in the medium. The enzyme activity in culture filtrate of different fungal strains was stable up to 7 days when stored at −20°C, and then after the activity declines. At the end of the 90 days, the enzyme activity from AL-3 remains about 6.8% of the original value when GG was used as carbon source. With WS the AL-3 enzyme was stable up to 30 days. WE-3, however, showed maximum enzyme stability up to 60 days when GG was used as a carbon source. Under this condition, the enzyme activity remained up to 34.3% of its activity on the seventh day. 

The fungal strain, AL-3, secreted maximum enzyme with GG as a carbon source with low level of stability where as WF-3 exhibited the potential to excrete the enzyme with greater level of enzyme stability as compared to that of AL-3. The results indicated that not only the extent of secretion of the proteins, enzyme and the stability of enzyme activity varied from one fungal strain to another but also their nature of utilization of different carbon sources.

## Figures and Tables

**Table 1:** Effect of different carbon sources on the activity of *α*-galactosidase secreted on the seventh day by different fungal strains.

Culture media (50 mL)	*α*-galactosidase activity (U/mL) secreted by different fungal strains			
Carbon sources (1%, w/v)	AL-3	WF-3	WP-4	CL-4
Guar gum (GG)	190.3 ± 8.09	174.5 ± 7.75	93.9 ± 5.53	28.8 ± 2.34
Wheat straw (WS)	41.5 ± 4.75	30.83 ± 3.32	59.1 ± 3.12	6.2 ± 0.37
Soya casein (SC)	63.5 ± 3.87	14.5 ± 0.76	37.4 ± 2.98	4.2 ± 0.21

The different fungal strains were inoculated into standardised culture medium containing different substrates, and the activity of *α*-galactosidase secreted into the culture filtrate was measured at the seventh day as described in Section 2. The results are presented as the mean value ± SD of three independent experiments.

**Table 2:** Total protein secreted by different fungal strains in culture filtrate at the seventh day by different fungal strains.

Culture media (50 mL)	Secreted protein (*μ*g/mL) by different fungal strains			
Carbon sources (1% w/v)	AL-3	WF-3	WP-4	CL-4
Guar gum (GG)	464.5 ± 11.09	59.1 ± 3.27	41.3 ± 3.67	13.8 ± 1.21
Wheat straw (WS)	230.3 ± 4.98	283.5 ± 6.39	265.7 ± 5.98	151.6 ± 4.58
Soya casein (SC)	59.1 ± 3.78	198.8 ± 4.22	47.3 ± 3.89	277.5 ± 6.08

The different fungal strains were inoculated into standardised culture medium containing different substrates, and the content of total protein secreted into the culture filtrate was measured at the seventh day as described in Section 2. The results are presented as the mean value ± SD of three independent experiments.

**Table 3:** Activity profile of *α*-galactosidase secreted by different fungal strains into culture medium containing three different substrates.

Incubation period in culture medium	Culture Media (50 mL)	*α*-galactosidase activity by different fungal strains			
		Residual activity (U/mL/month)			
	Carbon sources	AL-3	WF-3	WP-4	CL-4
7 days	Guar gum (GG)	190.3 ± 5.98	174.5 ± 4.78	93.9 ± 3.65	28.8 ± 1.32
	Soya casein (SC)	63.49 ± 2.45	14.5 ± 0.76	37.4 ± 1.43	6.2 ± 0.08
	Wheat straw (WS)	41.55 ± 1.66	30.8 ± 1.33	59.1 ± 2.12	4.16 ± 0.04

30 days	Guar gum (GG)	13.62 ± 0.56	76.2 ± 2.55	62.9 ± 2.13	25.3 ± 1.21
	Soya casein (SC)	20.61 ± 1.03	6.5 ± 0.06	32.6 ± 1.44	3.1 ± 0.04
	Wheat straw (WS)	41.38 ± 1.55	24.6 ± 1.10	36.9 ± 1.39	0.49 ± 0.01

60 days	Guar gum (GG)	12.96 ± 0.43	59.5 ± 2.11	25.4 ± 1.12	13.6 ± 0.34
	Soya casein (SC)	12.71 ± 0.39	6.3 ± 0.07	8.9 ± 0.08	2.9 ± 0.03
	Wheat straw (WS)	2.9 ± 0.02	24.3 ± 1.09	20.6 ± 1.02	0.49 ± 0.03

The different fungal strains were inoculated into standardised culture medium containing different substrates, and the activity of enzyme secreted into the culture filtrate was measured at varying periods of their culture as described in Section 2. The results are presented as the mean value ± SD of three independent experiments.
